# ATP directed agent, 8-chloro-adenosine, induces AMP activated protein kinase activity, leading to autophagic cell death in breast cancer cells

**DOI:** 10.1186/1756-8722-7-23

**Published:** 2014-03-14

**Authors:** Christine M Stellrecht, Hima V Vangapandu, Xiao-Feng Le, Weiqun Mao, Shujun Shentu

**Affiliations:** 1 Department of Experimental Therapeutics, University of Texas MD Anderson Cancer Center, Unit 1950, P.O. Box 301429, Houston, TX 77230-1429, USA; 2 Graduate School of Biomedical Sciences, The University of Texas Health Science Center, Houston, TX, USA

**Keywords:** Autophagy, AMPK, mTOR, ATP, 8-chloro-adenosine

## Abstract

**Background:**

8-chloro-adenosine (8-Cl-Ado) is a unique ribonucleoside analog which is currently in a phase I clinical trial for hematological malignancies. Previously, we demonstrated in breast cancer cells that a 3-day treatment with 10 μM 8-Cl-Ado causes a 90% loss of clonogenic survival. In contrast, there was only a modest induction of apoptosis under these conditions, suggesting an alternative mechanism for the tumoricidal activity of 8-Cl-Ado.

**Methods:**

Cellular metabolism, AMP-activated protein kinase (AMPK) and mammalian target of rapamycin (mTOR) pathway signaling, as well as autophagy induction was evaluated in breast cancer cell lines treated with 8-Cl-Ado. The effects of knocking down essential autophagy factors with small interfering RNA on 8-Cl-Ado-inhibited cell survival was assessed in breast cancer cells by examining apoptosis induction and clonogenic survival. *In vivo* efficacy of 8-Cl-Ado was measured in two breast cancer orthotopic model systems.

**Results:**

We demonstrate that in breast cancer cell lines, the metabolism of 8-Cl-Ado results in depletion of endogenous ATP that subsequently induces the phosphorylation and activation of the energy sensor, AMPK. This was associated with an attenuation of mTOR signaling and an induction of the phosphorylation of the autophagy factor, Unc51-like kinase 1 on Ser555. 8-Cl-Ado-mediated induction of autophagy was evident by increased aggregates of microtubule-associated protein 1 light chain 3B (LC3B) which was associated with its conversion to its lipidated form, LC3B-II, p62 degradative flux, and increased formation of acidic vesicular organelles. Additionally, transfection of MCF-7 cells with siRNA to ATG7 or beclin 1 provided partial protection of the cells to 8-Cl-Ado cytotoxicity as measured by clonogenicity. *In vivo*, 8-Cl-Ado inhibited growth of both MCF-7 and BT-474 xenograft tumors. Moreover, in 9 of 22 BT-474 tumors treated with 100 mg/kg/day 3 times a week, there was an absence of macroscopically detectable tumor after 3 weeks of treatment.

**Conclusions:**

Our data demonstrates that 8-Cl-Ado treatment activates the AMPK pathway leading to autophagy induction of in breast cancer cells, eliciting, in part, its tumoricidal effects. Additionally, 8-Cl-Ado effectively inhibited *in vivo* tumor growth in mice. Based on this biological activity, we are planning to test 8-Cl-Ado in the clinic for patients with breast cancer.

## Background

Breast cancer is the most prevalent cancer in women in industrialized countries. Each year more than a half million new cases of breast cancer are diagnosed in the US and Europe. Although in recent years, advances in therapies have contributed to decreased mortality; worldwide nearly 500,000 deaths are attributed to breast cancer, annually. In addition, toxicity associated with current treatments and the risk of developing secondary cancers [1] highlight the urgent need for new therapies against the disease.

8-chloro-adenosine (8-Cl-Ado) is a unique ribonucleoside analog that is currently in a phase I clinical trial at the UT MD Anderson Cancer Center for the treatment of chronic lymphocytic leukemia (CLL). Previously, we demonstrated this analog is tumoricidal to breast cancer cells [2]. In addition, our group and others have shown that 8-Cl-Ado inhibits the growth and survival of numerous other hematological and solid tumor model systems [3-19] but not normal lymphocytes [12] or non-transformed mammary epithelial cells (unpublished data).

Much of our prior work on the cytotoxic effects of 8-Cl-Ado focused on the accumulation of 8-Cl-ATP [2,8-10,12,20] and its incorporation into mRNA [10] preceding an inhibition of transcription [2,8-10,12,19] and poly(A) polymerase [21,22]. The net effects of these events lead to an induction of apoptosis [2,8-10,12]. In breast cancer cells, 8-Cl-Ado-induced cytotoxicity is only partially attributed to apoptosis. In addition to the impact on transcription, 8-Cl-Ado-induces a reduction of the intracellular ATP pool [2,8-10,12] and ATP depletion has been associated with apoptosis-independent cell death [23,24].

Within the cell, ATP levels are tightly regulated by AMP-activated protein kinase (AMPK), which functions as a cellular energy sensor [25]. AMP and ATP directly bind and regulate AMPK such that when ATP levels decrease, AMP is allowed to bind and allosterically activate AMPK. Additionally, upstream kinases can then phosphorylate and further activate AMP bound AMPK. Once activated, AMPK promotes events that lead to metabolic switches to restore cellular bioenergy. Significant energy regulating effects of AMPK pertaining to cancer therapeutics is its ability to attenuate growth and protein synthesis by regulating mammalian target of rapamycin (mTOR) pathway and induce autophagy.

AMPK inhibition of mTOR signaling occurs through two pathways. First, AMPK directly phosphorylates and activate tuberous sclerosis protein 2 which is involved in mTOR inhibitory signaling. Second, AMPK has been shown to phosphorylate and inactivate raptor, a member of mTORC1. Both mTORC1 and AMPK regulate autophagy through the phosphorylation of Unc51-like kinase 1 (ULK1). [For a review see ref. [26]]. ULK1 is a mammalian homolog of a yeast autophagy initiating factor, Atg1. Although there has been some discrepancy regarding the specific AMPK phosphorylation sites on ULK1, it is clear that AMPK activates ULK1 while the phosphorylation by mTORC1 inhibits ULK1 activity. AMPK also regulates autophagy through modulation of components of Vsp34 complexes [27].

The events above delineate the mode by which diminished ATP levels can alter cellular processes. Since 8-Cl-Ado treatment diminishes the ATP pool [2,8-10,12], we hypothesize that 8-Cl-Ado treatment will educe AMPK activation and mTORC1 attenuation which will initiate autophagy. Our current investigation further evaluated the cellular metabolism of 8-Cl-Ado and its metabolic effects on bioenergy. Moreover, we examined the mechanism of action of the bioenergy depletion that leads to a non-apoptotic cell death in breast cancer cells.

## Results

### 8-Cl-Ado accumulation/elimination and effects on energy production

**Figure 1:**
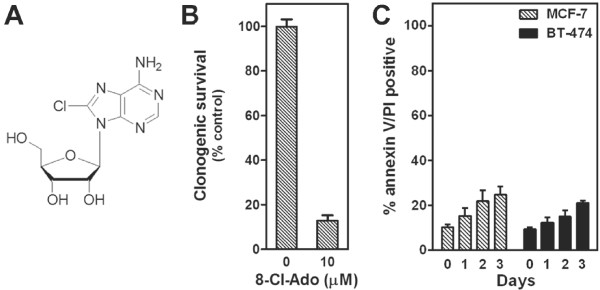
**Effect of 8-Cl-Ado on the survival of breast cancer cells. (A)** Structure of 8-Cl-Ado. **(B)** The effects of 8-Cl-Ado on the clonogenic growth of MCF-7 cells. Cells were treated with 10 μM 8-Cl-Ado for 3-days, washed with PBS, and cultured in fresh medium for 14-days. Colonies of >50 cells were counted under a dissecting microscope. **(C)** Flow cytometery analysis of annexin V and PI staining in MCF-7, *hatched bars*, and BT-474 cells, *black bars*, treated with 10 μM 8-Cl-Ado for the indicated times.

Previously, our group demonstrated that 8-Cl-Ado (Figure 1A) is tumoricidal to primary CLL lymphocytes [12], breast cancer [2], myeloma [17], and mantle cell lymphoma (MCL) [8] cell lines and this cytotoxicity was dependent on the analog’s metabolism to its monophosphate form by adenosine kinase [9] followed by triphosphate accumulation. In the breast cancer cell lines, we showed that a 3-day treatment with 10 μM 8-Cl-Ado inhibited over 90% clonogenic survival [2] (Figure 1B). Paradoxically, we demonstrated that such treatments were associated with only a ~30% induction of apoptosis in both cell lines as measured by annexin V and PI staining [19] (Figure 1C and Additional file 1: Figure S1).

**Figure 2:**
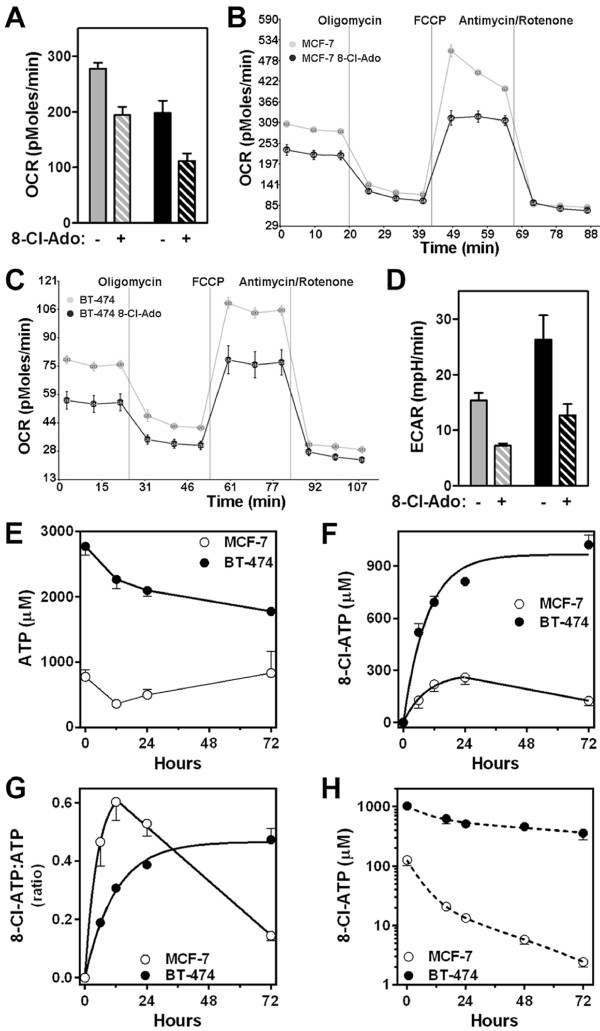
**Accumulation and elimination of 8-Cl-ATP and effects on ATP production. (A)** 10 μM 8-Cl-Ado treatment, *hatched bars*, perturbs basal OCR as compared to untreated, *solid bars*, in MCF-7, *gray bars*, and BT-474 cells, *black bars*. Effects of altering ETC complexes with the agents, oligomycin, FCCP, antimycin, and rotenone on OCR in **(B)** MCF-7 and **(C)** BT-474 cells treated with, *black circles*, and without, *gray circles*, 10 μM 8-Cl-Ado. **(D)** 10 μM 8-Cl-Ado treatment, *hatched bars*, reduces basal glycolysis as demonstrated by decreased ECAR in MCF-7, *gray bars*, and BT-474 cells, *black bars*. **(E)** Time dependent depletion of the endogenous ATP pool, **(F)** the accumulation of 8-Cl-ATP, and **(G)** changes in the 8-Cl-ATP/ATP ratio in MCF-7, ○, and BT-474 cells, ●. Cells were treated with 10 μM 8-Cl-Ado for the indicated times and acid extracts were analyzed by HPLC to measure nucleotide levels. **(H)** The elimination of 8-Cl-ATP in MCF-7, ○, and BT-474 cells, ●. Cells were treated with 10 μM 8-Cl-Ado for 3 days, washed with PBS, and cultured in fresh medium. At the indicated times, acid extracts were analyzed as above.

We established that 8-Cl-Ado treatment results in a rapid depletion of ATP within 12-hours which was associated with 8-Cl-ATP accumulation (Additional file 1: Figure S2 and [2]). To examine the impact of 8-Cl-Ado treatment on energy producing metabolic pathways, we analyzed both glycolysis and mitochondrial respiration. Extracellular acidification rate (ECAR), as a measure of glycolysis, and oxygen consumption rate (OCR), for mitochondrial respiratory function, were assessed in both MCF-7 and BT-474 cells treated with and without 8-Cl-Ado for 18 hr using a Seahorse XF96 analyzer. Our results indicated that, both basal mitochondrial respiration (Figure 2A, B, and C) and glycolysis (Figure 2D) were perturbed by 8-Cl-Ado treatment. The finding of decreased O_2_ consumption is in keeping with previous data which indicated 8-Cl-Ado may be an inhibitor of mitochondrial complex V, ATP synthase [28]. The alteration in glycolysis may possibly be associated with decreased glucose uptake, which was seen in myeloma cells treated with an 8-Cl-Ado congener compound, 8-amino-adenosine [29]. Interestingly, further assessment of cellular respiratory chain with XF Cell Mito Stress Test assay possibly revealed addition alterations in cellular respiration induced by 8-Cl-Ado treatment, as OCR was still attenuated even after the addition of an uncoupler, FCCP, to alleviate the dependency of complex I-IV on complex V’s transport of electrons across the mitochondrial membrane (Figure 2B and C). Because tumor cells are known to be sensitive to ATP depletion, we decided to assess the effects of 8-Cl-Ado on ATP levels throughout the 3-day treatment. Our results showed that in BT-474 cells the ATP levels continued to diminish over a 3-day treatment with 10 μM 8-Cl-Ado (Figure 2E) while in MCF-7 cells the levels remained below control levels but did show evidence of some recovery by 24-hours. Additionally, we examined the accumulation of the analog’s cytotoxic metabolite, 8-Cl-ATP, over 72-hours. The accumulation of the analog triphosphate inversely paralleled the ATP depletion as it peaked at 24- and 72-hours in MCF-7 and BT-474 cells, respectively (Figure 2F). Since the ratio of 8-Cl-ATP to ATP has been shown to be a determinant of the cytotoxic effect of 8-Cl-Ado [2,8], the kinetics of this relationship was also examined (Figure 2G). We demonstrated that the ratio of the analog triphosphate to normal ATP was highest in MCF-7 cells which rapidly peaked at 12-hours while in BT-474 cells the ratio continually increased until it reached a plateau by 24-hours. The elimination kinetics of 8-Cl-ATP was also measured by treating the cells with 10 μM 8-Cl-Ado for 3-days, washing drug off and continued culturing the cells in drug-free medium for another 3-days (Figure 2H). In both lines there appeared to be an initial, more rapid elimination of 8-Cl-ATP after drug removal. If a biphasic elimination kinetics is considered, the 8-Cl-ATP half-lives in MCF-7 cells were 3.8- and 25.5-hours while in BT-474 it was 6.4-hours and >7-days. An assessment of the kinetics as a monophasic elimination yields the 8-Cl-ATP half-lives as 5.8 and 11.4-hours for MCF-7 and BT-474, respectively.

### 8-Cl-Ado induces AMPK activity in breast cancer cells

**Figure 3:**
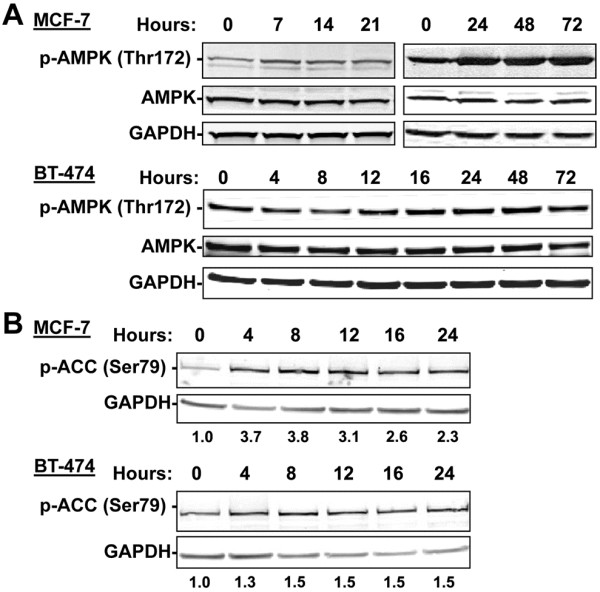
**Dose and time dependent induction of AMPK activity after treatment with 8-Cl-Ado.** Immunoblot analysis of phospho- and total AMPKα levels **(A)** in MCF-7 and BT-474 cells treated with 10 μM of 8-Cl-Ado for the indicated times. **(B)** Analysis of the phosphorylation levels of the AMPK target, ACC, in MCF-7 and BT-474 cells treated as described above. The normalized ratio of p-ACC to GAPDH relative to the untreated control is indicated beneath each lane. GAPDH was used as a loading control for all immunoblots.

The 8-Cl-Ado-induced ATP depletion is expected to increase the AMP to ATP ratio which would lead to activation of AMPK. To determine if 8-Cl-Ado is able to induce AMPK activity, we treated MCF-7 and BT-474 cells with 10 μM 8-Cl-Ado for various times and assessed changes in p-AMPK (Thr172) levels by immunoblot analysis. The results demonstrated that while total AMPK protein levels were unchanged by 8-Cl-Ado treatment, phosphorylation of AMPK (Thr172) was induced in a time-dependent manner, being readily detected within 7- to 12-hours (Figure 3A). To further assess AMPK activity, we examined the cellular phosphorylation of one of its critical downstream substrates, ACC on Ser79 and showed that AMPK activity is induced within 4-hours of 8-Cl-Ado treatment (Figure 3B). These events occurred in both MCF-7 and BT-474 cells which indicate they do not require p53 as MCF-7 cells have a wild type p53 genotype while BT-474 cells harbor a mutant p53 [30].

### Inhibition of mTOR by 8-Cl-Ado

**Figure 4:**
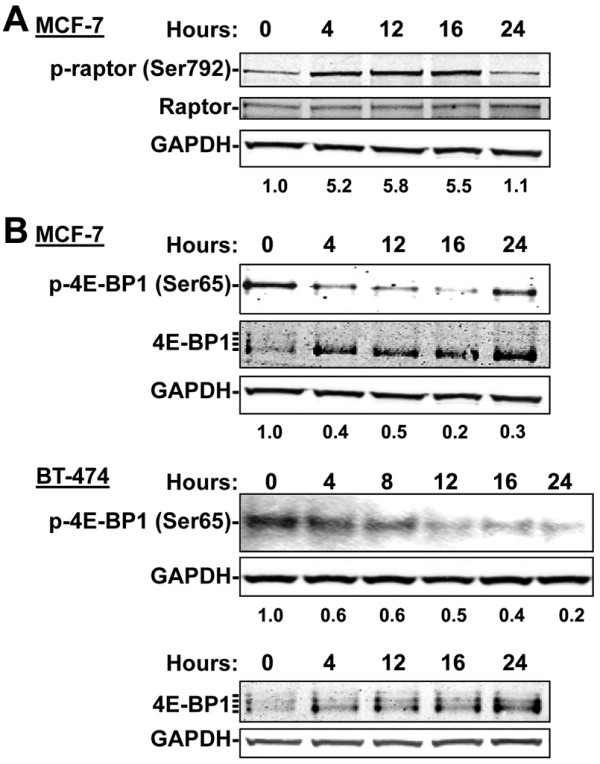
**8-Cl-Ado inhibits the activity of the mTOR pathway.** Immunoblot analysis of 8-Cl-Ado-induced **(A)** phosphoylation of raptor on the AMPK phosphorylation site, Ser792, as compared with total raptor and **(B)** specific inhibition of an mTOR phosphorylation site on 4E-BP1 Ser65 along with compression of slower migrating hyperphosphorylated 4E-BP1 bands in MCF-7 and BT-474 cells treated with 10 μM of 8-Cl-Ado for the indicated times. The normalized ratios of p-raptor Ser792 to GAPDH and of p-4E-BP1 Ser65 to total relative to the untreated control is indicated beneath their respective lane. GAPDH was used as loading controls for all blots.

A significant energy regulating effect of AMPK pertaining to cancer therapeutics is the ability of AMPK to inhibit the mTOR pathway. To determine if the 8-Cl-Ado-dependent AMPK induction altered the activity of the mTOR pathway, we assessed the effects of 8-Cl-Ado on Ser792 residue of raptor; an AMPK phosphorylation site on this mTORC1 protein. We determined that 10 μM 8-Cl-Ado-treatment readily induced the phosphorylation of raptor Ser792 (Figure 4A) in MCF-7 cells. This was associated with diminished mTOR autophosphorylation on Ser2481 (data not shown). To further assess mTORC1 activity, we examined the phosphorylation status of the mTORC1 target, 4E-BP1, and found 8-Cl-Ado treatment diminished the level of p-4E-BP1 (Ser65), a rapamycin sensitive phosphorylation site [31] and reduced the levels of the slower migrating hyperphosphorylated 4E-BP1 bands (Figure 4B). Overall, these results demonstrate 8-Cl-Ado treatment attenuated mTOR activity in breast cancer cells.

### 8-Cl-Ado-induced activation of ULK1

**Figure 5:**
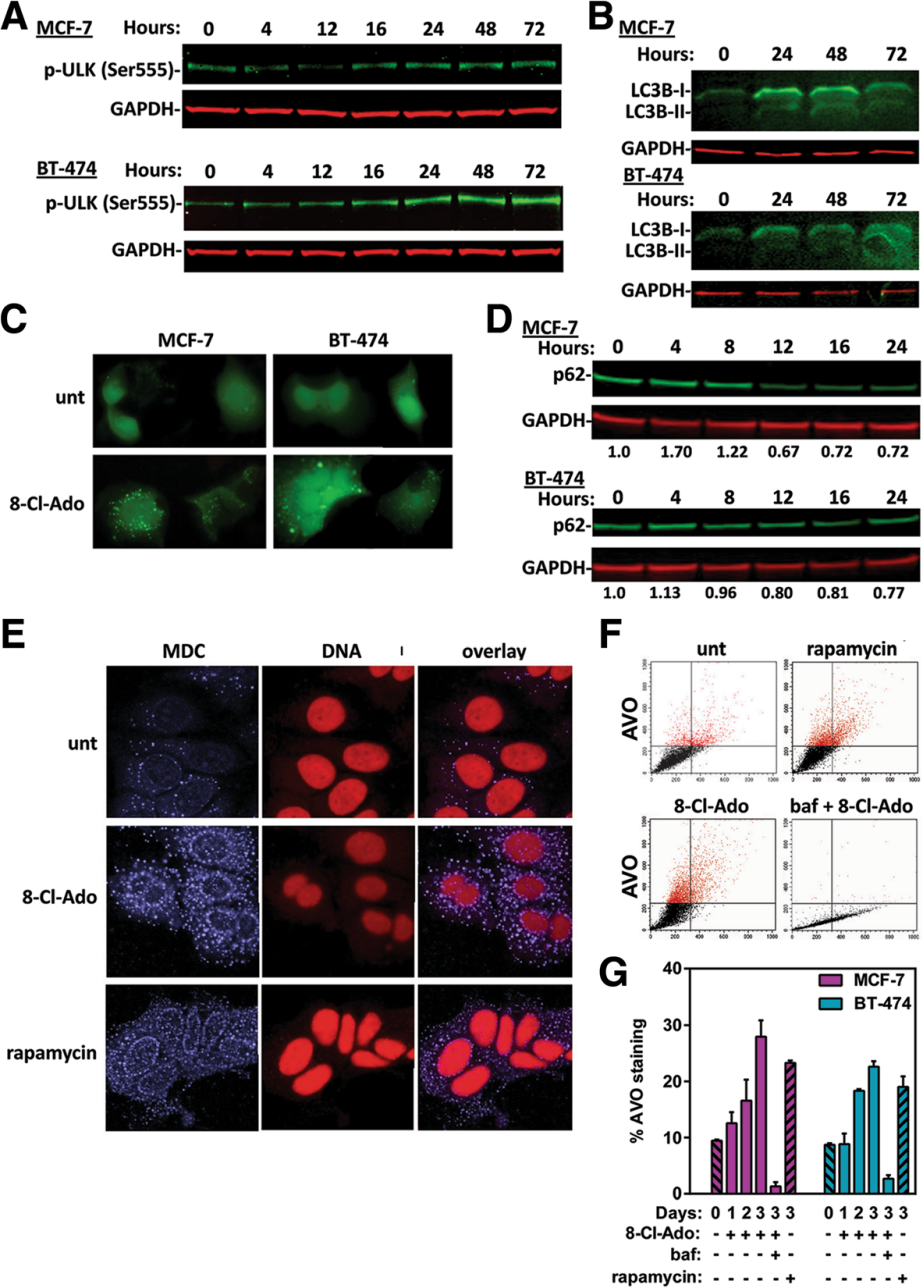
**8-Cl-Ado-induces autophagy.** Immunoblot analysis of **(A)** phospho-ULK (Ser555) levels and **(B)** LC3B-I lipidation to form LC3B-II in MCF-7 and BT-474 cells treated with 10 μM of 8-Cl-Ado for the indicated times. **(C)** Fluorescent microscopy of MCF-7 and BT-474 cells transiently transfected with GFP-LC3B and treated with 10 μM of 8-Cl-Ado for 12-hours to assess aggregation of LC3B. **(D)** Immunoblot analysis of p62 levels in MCF-7 and BT-474 cells treated with 10 μM of 8-Cl-Ado for the indicated times. The normalized ratio of p62 to GAPDH relative to the untreated control is indicated beneath each lane. **(E)** Fluorescent microscopic imaging of autolysosomes. MCF-7 cells were untreated or treated for 2-days with 8-Cl-Ado or rapamycin. Cells were stained with MDC, *blue*, to visualize AVO and Syto 61 (DNA), *red*, for nuclei counterstaining. **(F)** Representative flow cytometery histograms of AVO stained with acridine orange in MCF-7 cells untreated or treated for 3 days with 50 nM rapamycin or 10 μM 8-Cl-Ado. Baf was added before 30 min prior to staining to neutralize AVO staining. **(G)** Quantification triplicate experiments of MCF-7 and BT-474 cells treated and analyzed as in **E**.

AMPK and mTOR are both able to regulate autophagy through direct phosphorylation of Unc51-like kinase 1 (ULK1), which is the human counterpart of the yeast autophagy initiating factor, Atg1. Although there has been some discrepancy as to the ULK1 phosphorylation sites, there have been several different reports which indicate AMPK directly phosphorylates ULK on Ser555 [32-35]. We examined the effects of 8-Cl-Ado on the AMPK phosphorylation of this site and determined that 10 μM 8-Cl-Ado-treatment induces ULK1 phosphorylation on Ser555 in MCF-7 and BT-474 cells (Figure 5A).

### 8-Cl-Ado treatment of breast cancer cells elicits autophagy

Because both AMPK activation and mTOR inhibition can induce autophagy and 8-Cl-Ado treatment induced ULK1 Ser555 phosphorylation, we investigated whether 8-Cl-Ado would generate an autophagic response. To assess autophagosome formation, we used immunoblot analysis to evaluate lipidation of cleaved LC3B-I to the faster migrating autophagosome marker, LC3B-II. 8-Cl-Ado induced LC3B-I and LC3B-II protein levels in both MCF-7 and BT-474 cells (Figure 5B). Additionally, fluorescent microscopy of MCF-7 and BT-474 cells transfected with a LC3B-GFP expression construct, demonstrated that 8-Cl-Ado induced LC3B aggregation (Figure 5C), which further provides evidence of autophagosomes formation. Dynamic changes or flux in p62/SQSTM1 levels are also an indication of autophagy. This is due to the accumulation of p62 bound protein aggregates being sequestered during phagosome formation, followed by depletion because of autolysosomal degradation. 8-Cl-Ado treatment of MCF-7 and BT-474 cells induced an autophagic flux in both LC3B-II and p62 levels (Figure 5B and D).

Demonstration of autolysosome formation was performed by confocal microscopy of live MCF-7 cells stained with monodansylcadaverine (MDC) and syto61 which stains acidic vesicular organelles (AVO) and DNA, respectively (Figure 5E). The mTOR inhibitor, rapamycin, was used as a positive control. MDC staining of large AVO increased in 10 μM 8-Cl-Ado treated MCF-7 cells similar to treatment with 50 nM rapamycin. To quantitate the levels of AVO induction, 8-Cl-Ado treated MCF-7 and BT-474 cells were stained with acridine orange and assessed by flow cytometry (Figure 5F and G). Rapamycin and Bafilomycin A1 (Baf) treatment were used as positive and negative controls, respectively. As with the MDC staining, there is an induction of acridine orange staining of AVO in 8-Cl-Ado treated MCF-7 and BT-474 cells to levels similar to treatment with rapamycin. After 3-days of treatment, there were an 18 and 14% increase in AVO staining with 10 μM 8-Cl-Ado in MCF-7 and BT-474 cells, respectively which was comparable to the 14 and 10% increase seen with 50 nM rapamycin treatment over the same time. Similar results were seen in 8-Cl-Ado treated T47D, SK-BR-3, and ZR-75-1 cells (Additional file 1: Figure S3). Collectively, these results establish that 8-Cl-Ado induces autophagy in breast cancer cells.

### Depletion of autophagy proteins diminished 8-Cl-Ado induced cytotoxicity

**Figure 6:**
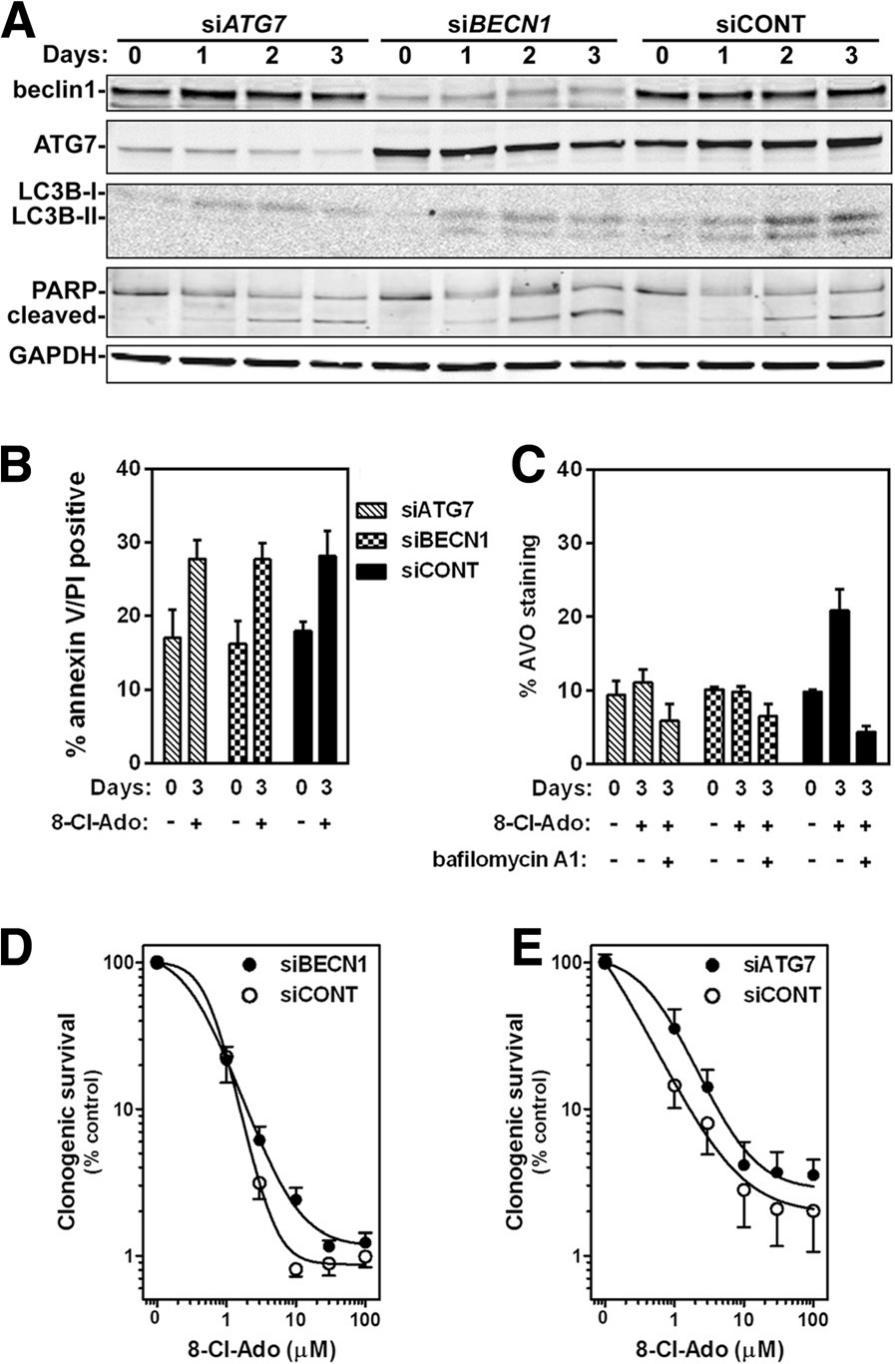
**8-Cl-Ado-induces autophagic cell killing. (A)** Western blot analysis of beclin1 and ATG7 levels in MCF-7 cells transfected with either a pool of control siRNA (siCONT), siRNA targeting the expression of the beclin1 gene (si*BECN1*), or targeting the expression of the *ATG7* gene (si*ATG7*). Immunoblot analysis of LC3B lipidation and PARP cleavage were assessed as markers of autophagosome formation and apoptosis, respectively*.* GAPDH was used as loading control. Flow cytometric analysis of cells transfected with siCONT, *solid bars*, si*BECN1*, *hatched bars*, or si*ATG7*, *checkered bars*, treated with 10 μM 8-Cl-Ado and stained with **(B)** annexin V and PI, as well as **(C)** acridine orange. Effect of autophagy on 8-Cl-Ado-inhibiton of clonogenic survival. Cells transfected with **(D)** siCONT, ○, or si*BECN1*, ●, and with **(E)** siCONT, ○, or si*ATG7*, ●, were treated with the indicated doses of 8-Cl-Ado for 3 days, washed with PBS, and cultured in fresh medium for 10 days. Colonies of >50 cells were counted under a dissecting microscope.

Previously, we had noted that with a 3-day treatment of 10 μM 8-Cl-Ado there was a ~90% loss of clonogenic survival while the amount of apoptosis induction only reached ~30% [2] (Figure 1B and C), suggesting there is another mechanism of cell killing occurring in the 8-Cl-Ado treated breast cancer cells. One possibility is through autophagic cell death. To test this hypothesis, we transfected MCF-7 cells with small interfering RNA (siRNA) directed against *ATG7,* or *BECN1*, which are required autophagy factors, and compared 8-Cl-Ado treatment of these cells to MCF-7 cells transfected with siCONT. The targeting siRNAs effectively depleted their respective targets during the 3 days of 8-Cl-Ado treatment and blocked autophagy induction (Figure 6A and C). Interestingly, although si*BECN1* and si*ATG7* did not alter the extent of 8-Cl-Ado-induced apoptosis (Figure 6A and B), they did increase clonogenic survival (Figure 6D and E). These results indicate that 8-Cl-Ado cytotoxicity is mediated in part by autophagic cell death.

### *In vivo* antitumor activity of 8-Cl-Ado in orthotopic breast cancer models

Our studies demonstrated 8-Cl-Ado is tumoricidal to breast cancer cells in cultures. To determine the efficacy of 8-Cl-Ado *in vivo* we established both MCF-7 and BT474 orthotopic tumors in nu/nu mice. Upon tumor formation, mice were treated for 3 weeks with varying doses up to 100 mg/kg/d 8-Cl-Ado 3d per week. Previous in cellular pharmacology analyses performed on peripheral blood mononuclear cells from CD_2_F_1_ mice after i.v. administration of 50 and 100 mg/kg 8-Cl-Ado, showed the 1 hr accumulation of 8-Cl-ATP was ~350 and ~1150 μM, respectively, [20] which was higher than the accumulation seen in the breast cancer cell lines treated with 10 μM 8-Cl-Ado [2], indicating tumoricidal doses are readily achievable. Additionally, an extensive toxicology assessment of numerous hematology, clinical chemistry, and microscopic pathology parameters of 8-Cl-Ado treatment in CD1 mice showed no toxicity at these doses [36].

**Figure 7:**
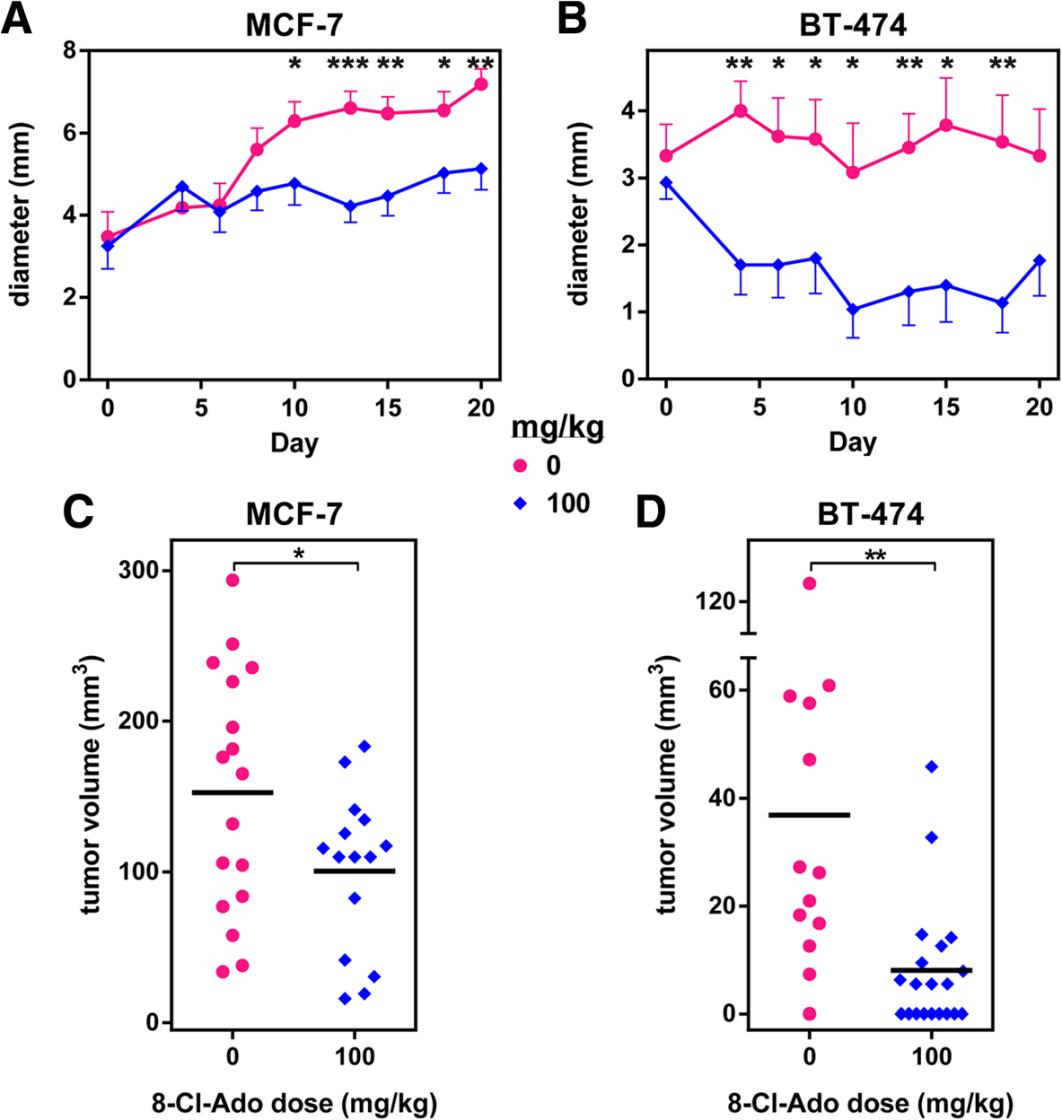
**Efficacy of 8-Cl-Ado in breast cancer xenograft models.** MCF-7 and BT474 xenografts in nude mice were established as described in Materials and Methods. Mice were treated with control PBS (0 mg/kg) or 8-Cl-Ado (100 mg/kg) three times a week for 3 weeks. MCF-7 **(A)** and BT-474 **(B)** tumor growth during 8-Cl-Ado treatment were assessed by measuring maximum tumor diameter each day of treatment. Final MCF-7 **(C)** and BT-474 **(D)** tumor volumes of tumors excised within 3 days of the final treatment. Statistical significance was determined using an unpaired *t*-test was used to, *P < 0.05, **P < 0.01, ***P < 0.001.

In the current study our results showed growth of the MCF-7 tumors were suppressed by the 100 mg/kg 8-Cl-Ado treatment (Figure 7A) which showed statistically significant differences by day 10 of treatment. Additionally, there was a dose dependent inhibition in a comparison of 0, 25, 50, and 100 mg/kg doses (data not shown). The growth of BT-474 tumors was dramatically altered as growth was significantly inhibited by the third day of treatment (Figure 7B). Furthermore, many of the tumors showed regression with the 100 mg/kg 8-Cl-Ado treatment. A 50 mg/kg dose did not affect the growth of the BT-474 xenograft tumors (data not shown). Similarly, an assessment of the final, excised tumor volume again showed mice treated with 100 mg/kg 8-Cl-Ado had statistically smaller MCF-7 and BT-474 tumor volumes after completion of the treatment (Figure 7C and D). Moreover, 9 of 20 BT-474 tumors completely regressed macroscopically. These results establish the potential for 8-Cl-Ado as a therapeutic agent to treat breast cancer and indicate BT-474 orthotopic tumors have a higher sensitivity to 8-Cl-Ado.

## Discussion

Previously, our investigations on the cytotoxic effects of 8-Cl-Ado focused on the accumulation of 8-Cl-ATP and its inhibitory effects on transcription [2,8-10,12]. In breast cancer cells, 8-Cl-Ado-induced cytotoxicity is only partially attributed to apoptosis. Depletion of the intracellular ATP pool has been associated with apoptosis-independent cell death [23,24]. Since 8-Cl-Ado treatment diminishes the ATP pool (Figure 2A) [2,8-10,12], the objective of the current study was to further evaluate 8-Cl-Ado metabolic effects on the cells and its involvement with non-apoptotic cell death in the breast cancer cell lines. Data presented in the present work indicates 8-Cl-Ado perturbs bioenergy production in breast cancer cells by altering both glycolysis and mitochondrial respiration. The resulting ATP depletion induces AMPK activation, leading to an attenuation of mTOR signaling and an induction of autophagic cell death.

Previous evidence suggested that the mode by which 8-Cl-Ado leads to ATP depletion is due to the analog’s metabolite, 8-Cl-ADP, being a substrate for ATP synthase for conversion to 8-Cl-ATP [28]. In that study, both the di and tri phosphate of 8-ClAdo were found to affect complex V, ATP synthase. Based on molecular modeling, 8-Cl-ATP was shown to fit well in the product site which is the “loose” binding conformation of F_o_F_1_-ATP synthase catalytic site, thus inhibiting catalysis of its natural substrate ADP to synthesize ATP. Our evaluation of basal mitochondrial respiration in breast cancer cells treated with 8-Cl-Ado is in agreement with this study’s finding on 8-Cl-Ado treatment inhibiting complex V activity.

Additionally, our data from the Mito Stress Test suggests 8-Cl-Ado also alters a component(s) of complex I-V of the electron transport chain complex (ETC) as well. This is in contrast to the prior study [28] which concluded 8-Cl-Ado treatment did not affect complex I-IV. Their premise was based on similar O_2_ consumption rate in cells treated with 8-Cl-Ado versus adenosine. While our analysis did not evaluate the effects of adenosine on these cells, our conclusions are based on the use of an uncoupler, FCCP, which allows complex I-V to work at full function even when complex V is inhibited. Under these conditions, the 8-Cl-Ado treated breast cancer cells still showed depressed rates of O_2_ consumption. One possible mechanism by which 8-Cl-Ado may be affecting the ETC is through disturbing the citric acid cycle. In MCL cells, 8-Cl-AMP was found to be metabolized to both 8-Cl-ATP and to succinyl-8-Cl-Ado [37]. The high metabolism to a succinylated moiety in MCL cells depleted fumarate, a component of the citric acid cycle. In MCL cells, the accumulation of succinyl-8-Cl-Ado was greater than 8-Cl-ATP. While high accumulation of succinyl-8-Cl-AMP was not seen in MCF-7 or BT-474 cells as the levels of succinyl-8-Cl-Ado reached less than 10% of the 8-Cl-ATP levels (unpublished data), it does suggests 8-Cl-Ado may have some degree of an effect on the citric acid cycle in breast cancer cells. Additional studies are ongoing to determine what component(s) of the ETC is affected by 8-Cl-Ado treatment.

Besides metabolism to succinyl-8-Cl-Ado, the analog may be indirectly affecting ATP production through altering glycolysis. An 8-Cl-Ado congener compound, 8-amino-adenosine has been shown to alter the localization and expression of glucose transporters, and reduce glucose consumption, in myeloma cells [29]. Thus, it probable that 8-Cl-Ado may also be altering glycolysis in breast cancer cells through similar mechanisms.

In the breast cancer cells, we found the 8-Cl-Ado-mediated depletion of the endogenous cellular ATP pool was associated with an induction of AMPK phosphorylation and activity as measured by phosphorylation of AMPK target proteins, ACC and raptor. In agreement with our work, both Han et al. [38] and Lucchi et al. [39] showed that 8-chloro-cyclic AMP (8-Cl-cAMP) also activates AMPK. Our group and others have demonstrated that 8-Cl-cAMP serves as prodrug for 8-Cl-Ado, as the cyclic analog is converted extracellularly in plasma or in medium to 8-Cl-Ado via the enzymatic actions of serum phosphodiesterase and 5′-nucleotidase [6,7,17,40]. In concert, studies using an adenosine kinase deficient cell line demonstrated that 8-Cl-cAMP needs to be converted to 8-Cl-Ado and metabolized to 8-Cl-AMP intracellularly to induce its cytotoxic effects [9]. In both studies by Han et al. [38] and Lucchi et al. [39], the authors went on to further examine the growth inhibitory effects of 8-Cl-cAMP-induced AMPK activity on p38 mitogen-activated protein kinase to induce apoptosis. Both groups conclusively demonstrated that 8-Cl-cAMP mediated AMPK and p38 activation promoted the growth inhibitory and apoptotic effects of 8-Cl-cAMP, as combinations with the p38 inhibitors attenuated these events.

It is also interesting to note that while the induction of AMPK and autophagy occurred in both MCF-7 and BT-474 cells, the induction was not as robust in the BT-474 cells. For example, phosphorylation of ACC reached 3.8 fold increase in MCF-7 and 1.5 fold in BT-474; flux in p62 levels in MCF-7 increased to 1.70 fold and then decreased down to 0.67 fold while in BT-474 increased to 1.13 fold and then decreased down to 0.77 fold change. MCF-7 and BT-474 cells are p53 wild type and mutant [30], respectively; thus, the occurrence of AMPK and autophagy induction in both cell lines indicates these events do not require p53. However, since there was less induction in BT-474 cells, this may suggest there might be some dependency on p53 to achieve the same level of induction as seen in MCF-7 cells. Though this notion is intriguing, additional studies would be needed to explore the possibility of p53 status playing a role in the differential induction levels. A study of 8-amino-adenosine in breast cancer cell lines MCF-7 and MDA-MB-231 (p53 mutant) cell lines also showed this analog was able to induce apoptosis and autophagy in both cell lines, though the mechanism for the autophagy induction was not examined [41]. Moreover, in agreement with our work, this study showed that inhibition of autophagy did not affect the level of apoptosis. Because clonogenic survival was not examined in this study, it is unclear if the inhibition of autophagy affected this survival.

Agents with AMPK agonist activity have generated considerable interest for use in cancer therapeutics [reviewed in [25]]. The diabetes drug, metformin, is believed to be an oxidative phosphorylation inhibitor that induces AMPK through its ability to decrease ATP levels. Metformin has been shown to suppress spontaneous tumor formation in various animal models as well as suppress *in vitro* and *in vivo* tumor growth. Moreover, several studies have shown metformin reduces cancer risks in diabetic patients as well as improved therapeutic response in those with breast cancer. Interestingly, *in vivo* studies in mouse model systems indicate both p53 deficient [42] and HER2 over expressing tumor cells [43] have an increased sensitivity to metformin treatment. Similarly, we demonstrated 8-Cl-Ado had the highest efficacy in the BT-474 xenograft tumors which are both p53 deficient and HER2 over expressing. While 8-Cl-Ado inhibited the growth of both MCF-7 and BT-474 xenograft tumors, 45% of the BT-474 tumors were no longer detectable macroscopically after a 3 week treatment with 100 mg/kg dose.

A study by Cheong et al. demonstrated that metformin in combination with the glycolysis inhibitor, 2-deoxyglucose, activated AMPK, inhibited mTORC1 and induced autophagy [23]. Furthermore, the authors stated that in 13 of 15 cancer cell lines tested, this combination was more cytotoxic than either agent alone. Moreover, the increased sensitivity of this couplet correlated with ATP depletion and was associated with the down regulation of the expression of key components of the ETC complex I. In contrast, combination of AICAR and 2-deoxyglucose was also able to activate AMPK and inhibit mTORC1 but was not cytotoxic and did not deplete ATP. This was attributed to increased expression of numerous genes in ETC I, II, III, IV and F_o_F_1_-ATP synthase complexes, which would promote ATP production. Although Cheong et al. did not evaluate autophagy in the AICAR treated cells, it is important to point out there is controversy as to whether AICAR promotes or inhibits autophagy [44-46]. Their results do indicate a superior therapeutic effect is achieved with a dual inhibition of energy pathways for targeting tumor bioenergetics. Based on our findings that 8-Cl-Ado is able to perturb glycolysis as well as deplete ATP it is interesting to speculate this ribonucleoside analog, as a single agent, may achieve the dual effects seen with metformin and 2-deoxy-glucose combination.

## Conclusion

In summary, we show that 8-Cl-Ado is cytotoxic to breast cancer cells and this cytotoxicity is mediated by both apoptosis and autophagy. The 8-Cl-Ado-induced depletion of ATP elicits the autophagic response through activation of AMPK and inhibition of mTORC1; with the endpoint of these events being the activation of an autophagy initiation factor, ULK1, leading to the induction of autophagy. This unique nucleoside analog is in a phase I clinical trial for hematological malignancies. Preliminary analysis of the cells from patients in the trial indicates ATP depletion, AMPK activation, and induction of autophagy occurs while the patients are undergoing treatment with 8-Cl-Ado (unpublished data). Taken together our results indicate that targeting the bioenergy production of breast cancer cells would be an effective strategy for treating this disease, which can be readily achieved with 8-Cl-Ado.

## Methods

### Materials

8-Cl-Ado was obtained from Dr. V. Rao at the Drug Development Branch of the National Cancer Institute and was dissolved in water. Rapamycin (LC Laboratories, Woburn, MA) and bafilomycin A1 (Baf) (Sigma-Aldrich, St. Louis, MO) were dissolved in DMSO.

### Cell culture, clonogenic assays, and transfection

MCF-7 and BT-474 cell lines were obtained from ATCC (Manassas, VA) and maintained in DMEM:F12 (Mediatech, Manassas, VA) supplemented with 10% fetal bovine serum in the presence of 5% CO_2_ at 37°C. Cells were routinely tested for *Mycoplasma* infection and were authenticated by short tandem repeat analysis by UT MD Anderson Cancer Center’s Characterized Cell Line Core facility. Colony formation assays were performed as described [2].

siRNA transfections were performed with ON-TARGETplus si*ATG7*, si*BECN1* SMARTpool or ON-TARGETplus siCONTROL (siCONT) (Dharmacon, Lafayette, CO). Dharmafect 4 was used to transfect 1.5 × 10^6^ MCF-7 cells with 600 pmoles siRNA as per the manufacturer’s protocol (Dharmacon). Expression knockdown was allowed to proceed for ~2-days before reseeding for treatment analysis. GFP-LC3 expression construct was obtained from Dr. Gordon Mills in the Department of Systems Biology, UT MD Anderson Cancer Center. Using Nucleofector Kit V, programs P-20 and T-20, respectively, (Amaxa Biosystems, Koeln, Germany), 2 × 10^6^ MCF-7 and BT-474 cells were transfected with 2 μg DNA. After transfection, cells were cultured for 1-day prior to selection with 500 μg/ml G418 > 4 weeks to obtain pools of stably transfected GFP-LC3 expressing cells.

### Measurement of glycolysis, oxygen consumption, and intracellular NTPs

OCR was measured using XF 96 Extracellular Analyzer instrument (Seahorse Bioscience Inc., Chicopee, MA). MCF7 and BT474 cells were plated at 30,000 cells per well on XF96 cell culture microplate in 100 μl of culture media. Cells were treated with 10 μM 8-Cl-Ado for 18 hrs. Media was then replaced with fresh XF assay medium (Seahorse Bioscience Inc.), supplemented with 17.5 mM Glucose and 2 mM Sodium Pyruvate, (175 μl/well) and is incubated in a CO_2_ free chamber of XF Prep station for 1 hr. XF Cell Mito Stress Test assay was performed as per the manufacturer’s instructions using the following final concentrations; 1.25 μM oligomycin, 1 μM FCCP, 0.75 μM antimycin and 1.25 μM rotenone (Seahorse Bioscience Inc.). Each assay was repeated at least twice. For ECAR, the culture media was replaced with glycolysis stress test media (prepared in accordance with Seahorse Glycolysis stress kit) supplemented with 2 mM of fresh L-Glutamine. For the glycolysis assay, all ports were injected with 25 μl of drugs for the following final concentrations; Port A-10 mM glucose, Port B −1.25 oligomycin, Port C– 100 mM 2-deoxyglucose.

Perchloric acid was used to extract NTPs from MCF-7 and BT-474 cells treated with 10 μM 8-Cl-Ado and neutralized extracts were analyzed by HPLC (Waters 600E System Controller; Waters Corp., Milford, MA, USA) as described [2].

### Flow cytometry

Analysis of annexin V and PI labeling was performed as described [2]. To detect and quantify the development of 8-Cl-Ado-induced AVO, treated and untreated MCF-7 and BT-474 cells were stained directly in culture with 1 μg/ml acridine orange (Invitrogen) for 15 minutes at 37°C essentially as described [47]. Thirty minutes prior to staining, 0.1 μg/ml Baf was added to a duplicate culture of 8-Cl-Ado treated cells as a control for negative AVO staining. Cells treated with 50 nM rapamycin were used as a positive control. Cells were removed from the plate with Accumax (Fisher Scientific) and combined with pellets of cells detached during treatment, then analyzed using a Becton Dickinson FACSCalibur flow cytometer and CellQuest software (San Jose, CA, USA).

### Immunoblot analysis

Exponentially growing cells were treated with 10 μM 8-Cl-Ado for various amounts of time and protein lysates were isolated and analyzed using an Odyssey Infrared Imaging System (LI-COR Biosciences) as described [19]. Primary antibodies were rabbit polyclonal antibodies against p-AMPKα (Thr172), AMPKa, p-acetyl-coA carboxylase (ACC) (Ser79), p-raptor (Ser792), p-mTOR (Ser2481), p4E-BP1 (Ser65) (Cell Signaling Technology), LC3B, beclin 1 (Novus Biologicals, Inc, Littleton, CO), p62 (Enzo Life Sciences, Farmingdale, NY); rabbit monoclonal antibodies against p-ULK1 (Ser555) clone D1H4, raptor clone 24C12, mTOR clone 7C10 (Cell Signaling Technology), ATG7 (Novus Biologicals, Inc, Littleton, CO); mouse monoclonal antibodies GAPDH clone 6C6 (Abcam, Inc, Cambridge, MA); and goat polyclonal antibody 4E-BP1 (Santa Cruz Biotechnology, Inc, Santa Cruz, CA).

### Microscopic labeling of autophagic vesicles

MCF-7 cells were seeded overnight into 4 well chamber slides at a density of 1.5 × 10^4^ cells/chamber followed by a 72-hours treatment with or without either 10 μM 8-Cl-Ado or 50 nM rapamycin. AVO were stained by incubating cells in 50 mM MDC (Sigma-Aldrich) in PBS at 37°C for 10 minutes and then washed three times with PBS. Nuclei were counter stained with 5 μM SYTO 61 (Invitrogen) in Tris-buffered saline (25 mM Tris, 150 mM NaCl, pH 7.5) at 37°C for 10 minutes and then washed twice with PBS and immediately analyzed at UT MD Anderson Cancer Center’s Flow Cytometry and Cellular Imaging Core Facility by fluorescence microscopy using an inverted microscope (Olympus 1X71, Melville, NY) equipped with a filter system (excitation filter: 350/50 nm, emission filter: 528/38 nm). Images were obtained with a Hamamatsu Orca II ER camera (Hamamatsu, Japan) and processed using the program Slidebook (3I, Denver, CO).

### Xenograft studies

MCF-7 and BT474 xenografts were established in 4- 6-week-old nu/nu female athymic nude mice (Department of Experimental Radiation Oncology Animal Facility, The University of Texas MD Anderson Cancer Center) supplemented with 0.72 mg 60-day release 17β-estrogen pellets (Innovative Research of America, Sarasota, FL) by inoculating 5 × 10^6^ MCF-7 cells or 1 × 10^7^ BT474 cells into two sites of the mammary fat pads [48,49]. When maximum tumor diameters reached ~3 mm, the animals were randomly allocated (8–10 mice per group) for a 3 weeks *i.p.* treatment with 8-Cl-Ado dissolved in PBS (0 or 100 mg/kg, 3 days/week). Body weight and tumor growth were assessed 3 days/week and maximum tumor diameters were recorded. Shortly after the end of the treatment (1–3 days), the mice were sacrificed, tumors were collected and tumor volumes were calculated in mm^3^ using the formula length × width × height × π/6. All experiments involving animals were performed in accordance with the guidelines of the Institutional Animal Care and Use Committee.

### Statistical analysis

All graphing, statistical, and regression analysis was performed using Prism software (GraphPad Software, San Diego, CA). The elimination of 8-Cl-ATP was examined by one phase and two phase decay nonlinear regression analysis.

## Abbreviations

8-Cl-Ado: 8-chloro-adenosine; 8-Cl-cAMP: 8-Cl-cyclic AMP; AMPK: AMP-activated protein kinase; AVO: Acidic vesicular organelles; Baf: Bafilomycin A1; CLL: Chronic lymphocytic leukemia; ECAR: Extracellular acidification rate; ETC: Electron transport chain complex; FCCP: Trifluorocarbonylcyanide phenylhydrazone; GFP: Green fluorescent protein; LC3B: Microtubule-associated protein 1 light chain 3B; MCL: Mantle cell lymphoma; MDC: Monodansylcadaverine; mTOR: Mammalian target of rapamycin; OCR: Oxygen consumption rate; p: Phospho; PI: Propidium iodide; siRNA: Small interfering RNA; Ulk1: Unc51-like kinase 1.

## Competing interest

The authors declare that they have no competing interest.

## Authors’ contributions

CMS wrote the manuscript, performed transfections experiments, immunoblot, HPLC, mouse xenograft, flow cytometry and microscopic analyses, analyzed and designed all experiments; HVP performed and analyzed glycolysis and aerobic respiration analysis; XFL and WM provided assistance and guidance in mouse xenograft studies; SS performed immunoblot and mouse xenograft analysis and prepared samples for HPLC and flow cytometry. All authors read and approved the final manuscript.

## Supplementary Material

Additional file 1: Figure S1 Effect of 8-Cl-Ado on the survival of breast cancer cells. **Figure S2**: Accumulation of 8-Cl-ATP and effects on ATP production. **Figure S3**: 8-Cl-Ado-induces autophagy. Click here for file
