# Supplementary material for: ATP directed agent, 8-chloro-adenosine, induces AMP activated protein kinase activity, leading to autophagic cell death in breast cancer cells
Source: J Hematol Oncol. 2014 Mar 14;7:23. doi: 10.1186/1756-8722-7-23 (PMC4007639; doi:10.1186/1756-8722-7-23)
Supplement: Additional file 1: Figure S1 — Effect of 8-Cl-Ado on the survival of breast cancer cells. Figure S2: Accumulation of 8-Cl-ATP and effects on ATP production. Figure S3: 8-Cl-Ado-induces autophagy. [file 1756-8722-7-23-S1.pdf]

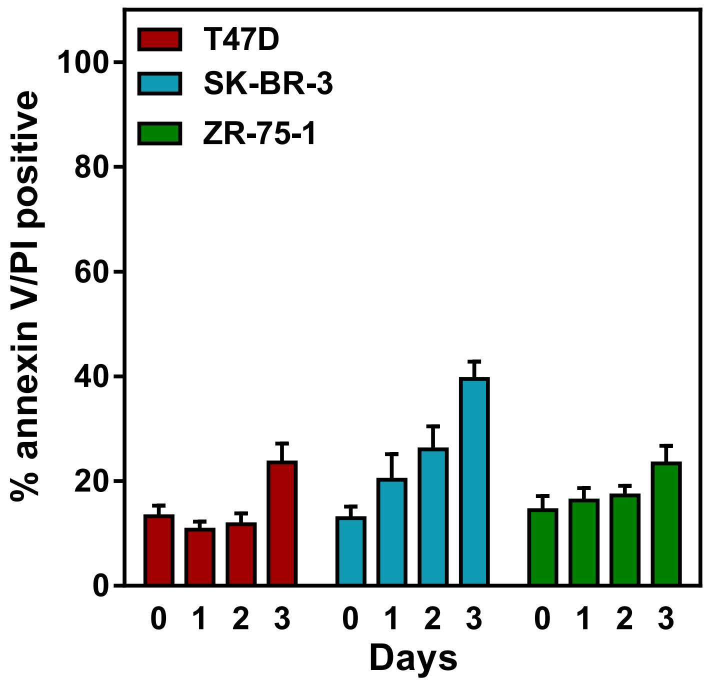

Figure S1 Effect of 8-Cl-Ado on the survival of breast cancer cells. Flow cytometery analysis of annexin V and PI staining in T47D, *dark red bars*, SK-BR-3, *teal bars,* and ZR-75-1 cells, *green bars*, treated with 10 μM 8-Cl-Ado for the indicated times.

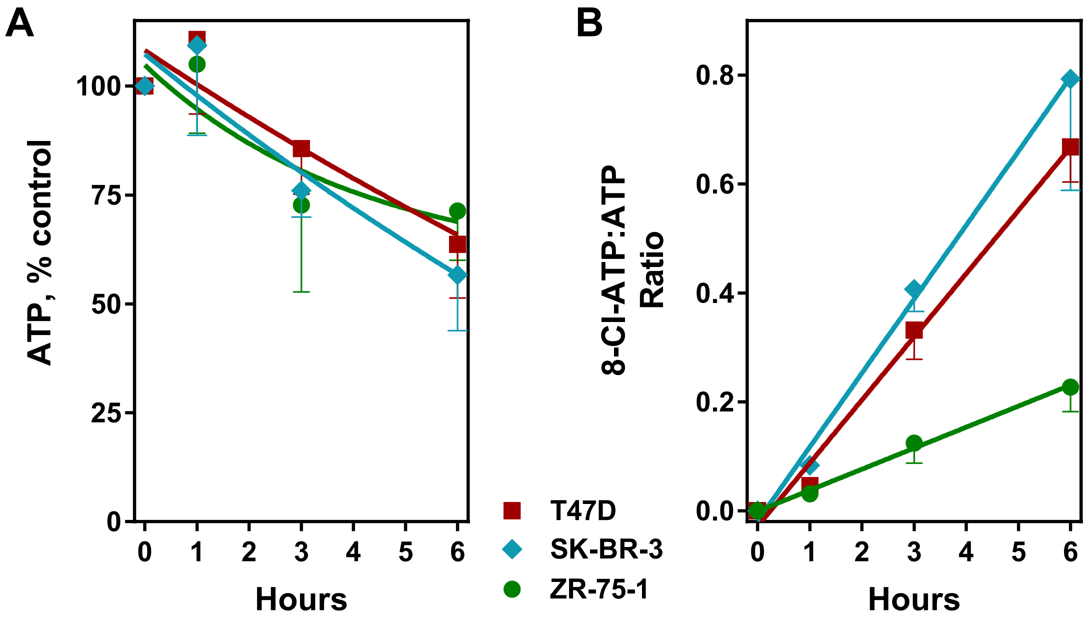

Figure S2 Accumulation of 8-Cl-ATP and effects on ATP production. (A) Time dependent depletion of the endogenous ATP pool and **(B)** changes in the 8-Cl-ATP/ATP ratio in T47D, ■, SK-BR-3, ♦, and ZR-75-1 cells, ●. Cells were treated with 10 μM 8-Cl-Ado for the indicated times and acid extracts were analyzed by HPLC to measure nucleotide levels.

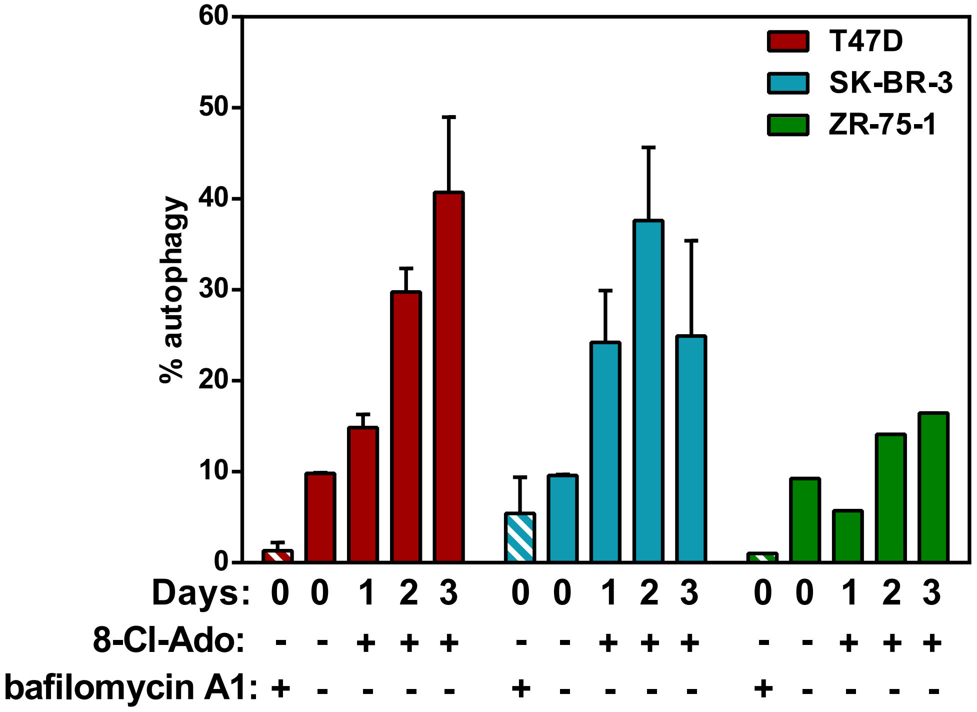

Figure S3 8-Cl-Ado-induces autophagy. Flow cytometery analysis of AVO stained with acridine orange in in T47D, *dark red bars*, SK-BR-3, *teal bars,* and ZR-75-1 cells, *green bars*, treated with 10 μM 8-Cl-Ado for the indicated times. Baf, *hatched bars* was added before 30 min prior to staining to neutralize AVO staining.
